# Understanding the Perspectives of Latino Adults Who Smoke on Physical Activity: A Qualitative Study

**DOI:** 10.3390/ijerph20043128

**Published:** 2023-02-10

**Authors:** Ruthmarie Hernández-Torres, Edgar Alaniz-Cantu, Maria Valeria Bautista Rojas, Daimarelys Lara, Sarah Merritt, Elisa DeJesus, Michelle Lee D’Abundo, Scott McIntosh, Deborah J. Ossip, Daniel Fuller, M. Patricia Rivera, Ana Paula Cupertino, Francisco Cartujano-Barrera

**Affiliations:** 1 Department of Public Health Sciences, University of Rochester Medical Center, Rochester, NY 14642, USA; 2 Health Literacy & Language Services, Ibero-American Action League, Inc., Rochester, NY 14605, USA; 3 Department of Interprofessional Health Sciences and Health Administration, Seton Hall University, Nutley, NJ 07110, USA; 4 School of Human Kinetics and Recreation, Memorial University of Newfoundland, St. John’s, NL A1C 5S7, Canada; 5 Department of Medicine, University of Rochester Medical Center, Rochester, NY 14642, USA; 6 Department of Surgery, University of Rochester Medical Center, Rochester, NY 14642, USA

**Keywords:** smoking, physical activity, Latinos

## Abstract

Smoking and sedentary lifestyle frequently co-occur among Latinos. Evidence suggests that moderate to vigorous physical activity (MVPA) may enhance smoking cessation rates. However, this synergistic phenomenon has not been studied among Latinos, the largest minority group in the United States. This qualitative study consisted of semi-structured interviews in English or Spanish with Latino adults who smoke (n = 20) to understand their perspectives on physical activity. Participants were recruited using community-based recruitment strategies. The Health Belief Model was used as a framework for qualitative theoretical analysis. Multiple perceived benefits (e.g., mood management, strategy to quit smoking), susceptibility (e.g., risk of cardiovascular diseases, physical impairment), and barriers (e.g., lack of social support, low financial resources) of being physically active were identified. Moreover, multiple cues to action to do physical activity (e.g., being a role model, spending time with family and friends) were identified. These factors provide concrete operational strategies to address smoking cessation and physical activity among Latinos. Further research is needed on how best to integrate these perspectives into smoking cessation interventions.

## 1. Introduction

Latinos, the largest minority group in the United States (U.S.), account for 17.4% of the current U.S. population and are projected to grow to approximately 30% by 2060 [1,2]. Of the nearly 55 million Latinos that reside in the U.S., over 4 million (8.0%) currently smoke cigarettes [3]. Latinos experience multiple barriers to healthcare access and treatment that result in tobacco-related disparities. Compared to non-Latino Black and white individuals, Latinos are less likely to receive advice to quit, participate in smoking cessation programs, and use pharmacotherapy or smoking cessation resources (e.g., quit lines) [4,5,6,7,8]. Specific barriers to accessing smoking cessation resources include language, literacy, mistrust of the health care system, and lack of knowledge about smoking cessation resources [9,10]. Latinos also report a general lack of cultural sensitivity within existing Spanish language resources for smoking cessation [11]. Although barriers to accessing smoking cessation resources exist, when provided with access, Latinos are interested in participating in smoking cessation programs and in using smoking cessation treatments to support quit attempts [12,13,14,15,16,17,18].

Over the past decade, research on the development and implementation of interventions for smoking cessation among Latinos has been conducted [13,14,15,16,17,18]. For example, one study assessed the feasibility and acceptability of a culturally and linguistically adapted smoking cessation text messaging intervention among Latino adults who smoke [15]. This study showed that such an intervention for Latinos offers a promising strategy to increase the use of pharmacotherapy (e.g., nicotine replacement therapies), generated high satisfaction, and resulted in noteworthy cessation rates at the end of treatment (30%) [15]. Despite the successes of these studies, to the best of our knowledge, no intervention has leveraged the role of physical activity in enhancing cessation rates among Latinos. Recent evidence suggests that moderate to vigorous physical activity (MVPA) may enhance cessation rates [19]. MVPA is associated with outcomes that predict quitting success, including acute relief from nicotine withdrawal, management of relapse/lapse, and greater self-efficacy to quit smoking [19,20,21,22,23]. MVPA also attenuates fear about post-cessation weight gain, a common concern among individuals who smoke [24,25,26,27,28]. Four studies have demonstrated higher cessation rates among individuals who smoke assigned to an MVPA intervention compared to the control group [25,26,27,28]. However, these trials were hampered by methodological limitations including no representation of minorities, which limits their generalizability to Latinos. The purpose of this qualitative study was to understand the perspectives of Latino adults who smoke on physical activity. Findings will be used to guide the development of an intervention that combines physical activity and smoking cessation.

## 2. Materials and Methods

### 2.1. Study Design

This qualitative study consisted of semi-structured interviews in English or Spanish with Latino adults who smoke to understand their perspectives on physical activity. Study procedures were approved and monitored by the University of Rochester Medical Center Institutional Review Board (protocol number STUDY00006996). Participants were compensated with a USD 30 gift card for their time and effort.

### 2.2. Recruitment 

Recruitment was conducted by a team of culturally diverse, bilingual (English and Spanish) trained recruiters between April and July 2021. Recruitment strategies included study presentations in venues with a high concentration of Latinos (e.g., community-based organizations, festivals, and malls) and word of mouth from participants and community partners. 

### 2.3. Eligibility 

Individuals were eligible if they (1) self-identified as Hispanic and/or Latino; (2) spoke English and/or Spanish; (3) were ≥21 years old; (4), on average, smoked cigarettes at least three days per week; and (5) were comfortable with communicating over Zoom^®^ or a phone call for the interview. Study staff conducted an eligibility assessment over the phone in the participant’s language of preference, either English or Spanish.

### 2.4. Consent 

Individuals eligible to participate in the study received an informational letter with detailed information about the purpose of the study, potential risks and benefits, incentives, study procedures, voluntary participation and the right to withdraw, confidentiality, data management, and the contact information of the study team. The informational letter was available in English and Spanish. The first and last authors reviewed the informative letter with participants and allowed them to ask questions before agreeing to participate in the study. Verbal consent was obtained from the participants before enrollment in the study.

### 2.5. Data Collection

Before the interview, the first and last authors conducted a sociodemographic survey used to collect information on age, gender, sexual orientation, employment status, the language of preference, and country of birth. Moreover, smoking-related variables were collected, including the number of cigarettes smoked per day (CPD), time to the first cigarette, and use of menthol cigarettes. The survey was completed in the participants’ language of preference, either English or Spanish. In previous studies, the sociodemographic and smoking-related questions have been used among Latinos who smoke [13,14,15,16,29].

Immediately after the sociodemographic survey, the first and last authors conducted a semi-structured interview. An interview guide with a list of open-ended questions was used to facilitate the interviews. The interview was completed in the participants’ language of preference, either English or Spanish. The Health Belief Model (HBM) was used to develop the interview guide. The HBM posits that people act to change their health behaviors (e.g., being physically active) based on their perceived benefits, susceptibility, barriers, cues to action, and self-efficacy [30]. *Perceived benefits* refer to a person’s opinion of the value or usefulness of a new behavior in lowering the risk of a disease and/or condition [30]. *Perceived susceptibility* refers to a person’s belief about their chances of getting a certain disease and/or condition [30]. *Perceived barriers* are a person’s view of the obstacles that stand in the way of behavior change [30]. *Cues to action* are events, people, or things that trigger people to change behavior [30]. *Self-efficacy* is a person’s confidence and belief in ability to take action or perform a given behavior [30]. Table 1 shows examples of the interview questions by the HBM factor.

The interviews were conducted by the first and last authors. All interviews were audio-taped and subsequently transcribed verbatim in the language in which they were conducted by the second, third, and fourth authors. Additionally, interviews in Spanish were translated into English by the third and fourth authors. The translations were verified by the interviewers, and any substantial discrepancies were resolved using a consensus approach. 

### 2.6. Analyses

For the sociodemographic and smoking-related questions, means and frequencies were calculated. For the interviews, qualitative theoretical analysis was used to identify, analyze, and report themes within the data [31]. The second and third authors coded the first five transcripts independently through line-by-line coding. An iterative process was employed to achieve consensus between the two sets of codes, create a coding scheme, and develop a codebook that included details about agreed-upon code definitions. New codes were added as needed to the coding scheme until no new themes emerged with successive interviews [32]. The last author supervised the qualitative theoretical analysis and provided in-depth feedback on the codebook development. All authors agreed on the final themes and sub-themes.

### 2.7. Positionality 

In qualitative studies, the researcher is considered a research instrument given their ability to observe details, conduct in-depth interviews, and reflect on the meaning of observations and interview data [33]. The first and last authors, who conducted the semi-structured interviews, are Latino researchers trained in community-based participatory research to address tobacco-related disparities among Latinos. The second and third authors, who conducted the qualitative theoretical analysis under the supervision of the last author, are Latino medical students interested in promoting Latino health.

## 3. Results

At baseline, the participants’ mean age was 54.9 years old (SD 12.1), 50% of the participants were female, 85% self-identified as heterosexual or straight, and 25% were employed full-time (Table 2). Eight participants (40%) indicated their language of preference as “Only Spanish,” and 50% were born in Cuba or the Dominican Republic (25% for each country). Most participants (70%) smoke 1–10 cigarettes daily, 35% smoke their first cigarette within five minutes after waking up, and 50% use menthol cigarettes. Table 3 describes each participant’s age, gender, and language of preference.

**Figure 1:**
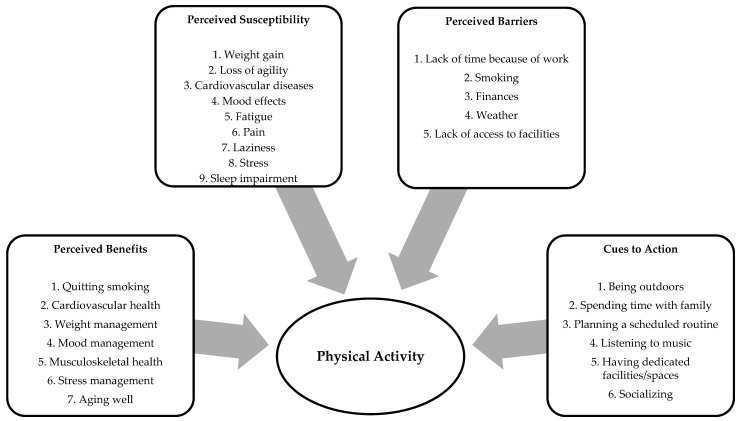
Perceived benefits, susceptibility, barriers, and cues to action to do physical activity among Latinos who smoke.

Multiple perceived benefits, susceptibility, barriers, and cues to action to do physical activity were identified. Figure 1 illustrates the perspectives of Latino adults who smoke on physical activity.

### 3.1. Perceived Benefits

Participants identified seven benefits of being physically active: (1) quitting smoking, (2) cardiovascular health, (3) weight management, (4) mood management, (5) musculoskeletal health, (6) stress management, and (7) aging well. Select quotes from participants are shown below. 

*Quitting smoking*: Fourteen participants described that physical activity was beneficial for quitting smoking:

“Well, I think it [physical activity] would be good, because, for example, instead of lighting up a cigarette, I’ll say, ‘No, I’m going to go do a little bit of something, some sort of exercise in the bedroom’. So that I forget, stop smoking. If I go to the bedroom and do a little bit of exercise, I’ll forget.”

Participant 4

“I think it [physical activity] would be to have something to distract yourself because I, in my case, smoke because of nerves, loneliness, tension. I don’t have to smoke because... because if I’m distracted, I don’t smoke.”

Participant 5

“Gradually [after being physically active], you’ll see that you’ll either start slowing down the smoking, because you’re thinking more healthy-wise, physically-wise.”

Participant 17

*Cardiovascular health.* Nine participants noted that being physically active was beneficial for the cardiovascular system:

“It [physical activity] is good for keeping the body oiled.”

Participant 2

“Physical activity helps you with the circulation.”

Participant 3

*Weight management.* Eight participants described that weight management was a benefit of being physically active:

“It [physical activity] also helps you maintain your weight. Not only does it help you with your body, but it also helps you maintain your weight… Exercise has many benefits.”

Participant 3

*Mood management.* Six participants noted that mood management was a benefit of being physically active:

“Yeah, [when being physically active], I feel lighter, like more relaxed, in the mood to do other things, I feel like... Like lighter, motivated.”

Participant 4

“Physical activity keeps me busy and I forget about depression… For example, physical activity releases my anxiety...”

Participant 12

*Musculoskeletal health*. Six participants described that physical activity has a positive impact on the musculoskeletal system:

“Because when you move and do physical activity, well, the muscles, the... The... Everything, everything moves and you feel better.”

Participant 5

*Stress management.* Five participants noted that stress management was a benefit of being physically active:

“Yes. I want to say that exercise is very good for people because you get out stress, any bad thoughts that come into your mind they kind of go away. ”

Participant 4

“Because you have to, that is, you go to the park [to do physical activity] and detox for pure... Doing exercise one sweats… I feel relaxed because I have no tension about anything…”

Participant 5

*Aging well.* Five participants described that aging well was a benefit of being physically active:

“You can live longer, so that your body feels better, so that you can live happily... More... With more energy. That motivates me, I feel that my body is moving better, that the years are passing but I still have that energy, that willingness to live, having a willingness to live, willingness to be healthy.”

Participant 3

“Well, I say, you know, physically, doing anything you can do is... To me, you know, it helps your mind to keep you young and you know, and mind-wise, you keep your body to doing things that you feel you can’t do no more....”

Participant 7

### 3.2. Perceived Susceptibility

Participants identified nine susceptibilities of not being physically active: (1) weight gain, (2) loss of agility, (3) cardiovascular diseases, (4) mood effects, (5) fatigue, (6) pain, (7) laziness (8) stress, and (9) sleep impairment. Select quotes from participants are shown below.

*Weight gain.* Nine participants noted weight gain as a susceptibility to not being physically active:

“If you don’t exercise, you get more... You gain weight... And you feel bad you gained weight...”

Participant 15

*Loss of agility.* Eight participants described loss of agility as a susceptibility to not being physically active:

“Well… Like a car when it stops moving, it gets rusty... And that’s how human beings are, that’s how human beings are, we lose our entire body’s mobility. Because sitting 24/7 is not healthy, it’s not healthy for anyone.”

Participant 5

*Cardiovascular diseases.* Three participants noted cardiovascular diseases as a susceptibility to not being physically active:

“Well, people in general, when they don’t do physical activity, well, they tend to get obese, they get diabetes, they get high blood pressure, their veins get clogged. Well, countless things… Many… And more so when a person smokes. Heart problems, diabetes, high blood pressure, cardiac arrhythmia. Well, anything can happen.”

Participant 13

*Mood effects.* Six participants described fatigue as a susceptibility to not being physically active:

“When you’re not moving, you’re thinking about everything. All the bad things, because it’s none of the good things... My mind is always in constant motion, and thinking, and thinking, and thinking, and it’s a… Sometimes it depresses you, you get depressed because you are there doing nothing… You are discouraged all the time. Yes, there are days when you get up and you’re happy and you do this and that, but I think that a person who is in constant movement does not feel so much… So much depression...”

Participant 3

“It [not being physically active] would cause me anxiety, it would cause me discomfort in my body, it would cause me a number of things. Depression, because I am not busy. But physical activity keeps me busy and I forget about depression. Because my disability is mental. Acute depression and anxiety problems and the.... Panic attacks...”

Participant 12

“When I don’t do anything [any physical activity] all day, I get a little depressed… I get very sad all I want to do is to go to bed and go to bed and sleep and sleep, as if to forget everything”

Participant 19

*Fatigue.* Six participants noted fatigue as a susceptibility to not being physically active:

“...When you don’t do physical activity you feel like your body is tired.”

Participant 16

*Pain.* Five participants described pain as a susceptibility to not being physically active:

“When I don’t exercise, the pain in my leg and hands is quite strong.”

Participant 11

“When I don’t do any [physical] activity, my whole-body hurts. My whole body hurts.”

Participant 15

*Laziness*. Three participants noted laziness as a susceptibility to not being physically active:

“Well, when one doesn’t engage [in physical activity], well one practically gets like, like I am, lazy. No desire to do anything”

Participant 16

*Stress*. Two participants described stress as a susceptibility to not being physically active:

“The stress. Here many... Here most people have stress and you have to focus in that direction [of being physically active] to eliminate stress and feel good.”

Participant 18

*Sleep impairment*. Two participants noted sleep impairment as a susceptibility to not being physically active:

“If I don’t exercise, if I don’t go for a walk at least once a day, then I’m not tired enough to go to sleep at night. I stay up.”

Participant 19

### 3.3. Perceived Barriers

Participants identified five barriers to being physically active: (1) lack of time because of work, (2) smoking, (3) finances, (4) weather, and (5) lack of access to facilities. Select quotes from participants are shown below.

*Lack of time because of work.* Thirteen participants described that lack of time because of work was a barrier to being physically active:

“My work, at one point, I had three jobs. And that was real bad. I wouldn’t be... All I did was go to work, come home for an hour, go back to work. And then come straight to bed. And then, when the middle of the week would come around, I would come from work, stay home for an hour, go back to work, and then had the other job. So that... It was tiresome. I was beating myself up but a bad way. I was killing myself “con tres trabajos” [with three jobs].”

Participant 1

“[Lack of] time... Look, I think it is laziness and the desire not to do it and also lack of time because, for example, I get home [from work] at 7 or 7:30 at night. I feel lazy to exercise at that time.”

Participant 11

“What I need is time… The only day I have free… I work from Monday to Saturday. The only day that I could do an exercise routine is on Sunday, when I don’t work.”

Participant 13

“Well, the [work] schedule, the [work] schedule. For example, I come in very early to work. When I come back [home], I come back very tired. ”

Participant 18

*Health constrains.* Ten participants noted health constrains as barriers to being physically active:

“It is difficult for me [to be physically active] because of my knees, because of my hands, in which I have had a carpal tunnel for many years, things fall out of my hands. My spine, I have a lot of problems with my spine, and fibromyalgia that doesn’t let me live in peace...”

Participant 5

*Smoking*. Nine participants noted that smoking was a barrier to being physically active:

“Well, a lot of them, the ones who smoke a lot—like I used to smoke—it’s just like a laziness that tobacco causes you. Since usually breathing is not optimal, you prefer not to [be physically active]. Because it’s not the same… Having your lungs pure than when you’re smoking. You know, you’re less dynamic, you’re more prone to sitting than exercising… For those who smoke a lot. Because that’s how it is for me sometimes.”

Participant 2

“But since I started smoking again two years ago—because I had quit smoking for almost 9 months because I was pregnant—I’ve seen the change. Horrible. The change is from heaven to earth. I get tired going up the stairs. If I’m talking to someone and we’re having a really quick conversation or something, I have to be gasping for air. If I do... If I jump a little, I do a little exercise, I can’t breathe… My chest is tight. The difference is quite big.”

Participant 3

“…People [who smoke] get very tired. When people smoke and they start exercising, they get tired, because the smoke is already in your system, and when you start doing exercise, you get tired over nothing. That happens to me, I get tired from nothing, I get out of breath.”

Participant 4

“Because it takes a lot, because well when I started doing that [physical activity] it was very hard… I suffocated very quickly, I got tired very quickly. I mean, like it takes you another… A little more to exercise.”

Participant 6

“Not having the physical condition due to lack of air from smoking.”

Participant 19

*Financial.* Three participants described financial problems as barriers to being physically active:

“Well, economic problems… Because what I... What I earn doesn’t cover anything. I would need money to pay for the gym.”

Participant 12

*Weather.* Three participants noted the weather as a barrier to being physically active:

“No, I haven’t started [going on walks with friends] yet because of the cold... No, currently I’m not doing them. But before, I used to like exercising a lot. Right now, I’m a little lazy because of the cold.”

Participant 4

*Lack of access to facilities*. Two participants described that lack of access to facilities was a barrier for being physically active:

“Well, I think [I am not physically active] because I’m not where I used to live a few years ago. Before I had the facilities right there where I lived. A basketball courts.”

Participant 2

### 3.4. Cues to Action

Participants identified six cues to action to being physically active: (1) being outdoors, (2) spending time with family, (3) planning a scheduled routine, (4) listening to music, (5) having dedicated facilities/spaces, and (6) socializing. Select quotes from participants are shown below.

*Being outdoors*. Ten participants noted that being outdoors was a cue for being physically active:

“I used to go to the parks a lot or go nature walking, you know. Like being out in, you know, in the woods and stuff like that. Taking all of nature, you know, enjoying nature. So, I used to do a lot of walking and stuff like that. Like I said, I used to ride my bicycle all over the place. I would go down to [local towns in upstate New York] on my bicycle, you know, go to the trails.”

Participant 7

*Spending time with family.* Seven participants described that spending time with family was a cue for being physically active:

“My niece has already finished university. She has already graduated, but I want to see her enter, because she wants to enter medical school. I would like to die when she is already a doctor… And so that’s why I want to be physically active.”

Participant 13

*Planning a scheduled routine.* Six participants noted that planning a scheduled routine was a cue for being physically active:

“Well, time. Planning your time. What time you’re [doing it], if you have time available. That is, set a program, organize yourself. That’s all you need. Because the [fitness] shops are there and the road [to go to a facility] is there. Everything is there, but it is getting organized…”

Participant 2

*Listening to music*. Five participants described that music was a cue for being physically active:

“Music is the way you can do whatever you want to do. I put music to clean, I put music to cook, I put music to shower, everything. Because if you want to move, the music you like—not just any music, the one you like—is going to make you feel different, it makes you feel different, and you’re going to want to dance, you’re going to want to move. And without you realizing it you are going to exercise, and a lot...”

Participant 3

*Having dedicated facilities/spaces.* Five participants noted that having dedicated facilities/spaces was a cue for being physically active:

“Well, a home gym will be fine, will be great. Cause you know, as long as you have the space... Okay, for myself, I have like the bench with some weights. Okay, I have that. I have a stationary bicycle, but that’s all I have right there. I mean, stretching bands are good, but I don’t have those. A pull up bar will be nice to put down there, but I don’t have that neither. And the, you know, a mat. Get a couple of mats so you can do push-ups. Or even put a rug downstairs, do the push-ups is good and you could do sit-ups. Crunches.”

Participant 1

*Socializing*. Four participants described that socializing was a cue for being physically active:

“Well… I would like to go to the gym. In the gym, I can hang out with other people...”

Participant 4

## 4. Discussion

This qualitative study was focused on the perspectives of Latino adults who smoke on physical activity using semi-structured interviews. The HBM was used as a framework for interview guide development and qualitative theoretical analysis, facilitating the understanding of perceived benefits, susceptibility, barriers, and cues to action. Study findings may help healthcare providers and researchers develop or adapt existing interventions to promote smoking cessation and physical activity among Latinos. 

In 2012, Strong et al. conducted a qualitative study to understand barriers and facilitators of physical activity among Latinos of Mexican origin who smoke [34]. Findings from our study expand prior research by understanding the perceived benefits, perceived susceptibility, and cues to action to physical activity. In addition, our study consists of a diverse sample of Latinos regarding country of birth: only 10% of participants are of Mexican origin. Nevertheless, the results of this study confirm two previously described barriers: (1) work schedule and/or activities leave little time and/or energy for being physically active, and (2) health problems limit participants’ ability to be physically active [34]. Future interventions for Latinos may be more effective when planned with attention to the perceived barriers identified in this study. For example, increasing physical activity could be studied as part of daily living activities: Work (e.g., attending walking meetings), household activities (e.g., sweeping and mopping the floor), transportation (e.g., walking to work or the bus stop), and recreational activities (e.g., walking the dog). Furthermore, interventions to increase physical activity could use the Physical Activity Readiness Questionnaire (PAR-Q) as part of the eligibility assessment to reduce the risk of injury. The PAR-Q is a screening assessment designed to determine the safety of doing physical activity for an individual based on their health history, current symptoms, and risk factors [35].

In this study, participants identified finances as a barrier for being physically active. This perception has been described among Latinos (regardless of their smoking status) in other studies [36]. It is imperative that interventions that promote physical activity among Latinos address this perceived barrier. For example, interventions should emphasize physical activities that are free of cost (e.g., walking, running). Participants identified lack of access to facilities as another barrier for physical activity. Carlson et al. showed that Latino adults living in neighborhoods with access to supportive walking and recreational facilities (e.g., parks) reported more physical activity than those living in areas less supportive of walking and recreational activity [37]. Therefore, future interventions may be more impactful by considering neighborhood environments when promoting physical activity among Latinos. For example, interventions could outline that in the absence of access to facilities, individuals can do physical activity at work and/or home (e.g., exercise programs with little or no equipment needed).

Participants identified smoking as a barrier to physical activity. According to participants, smoking is associated with shortness of breath when performing physical activities. Epidemiological studies have shown that cigarette smoking is one of the greatest risk factors for most respiratory symptoms, including dyspnea [38]. Fortunately, these studies have also shown that smoking cessation reduces shortness of breath over time. Importantly, participants noted that being physically active could facilitate smoking cessation and improve mental and physical health. These perceived benefits have been described in previous studies among Latinos) [39,40]. 

Participants identified weight management as a perceived benefit of physical activity. Evidence suggests that physical activity is a less important factor in weight loss or maintenance than diet, and that to achieve weight management 300 min of moderate to vigorous physical activity is recommended [41]. Interventions to increase physical activity should limit their focus on weight management, as it can make individuals feel that physical activity is not improving their health if they are not losing weight [42]. Interventions should focus on the fact that independent of weight, physical activity is a good activity for everybody [43,44].

Participants in our study described spending time with family as a cue to action. Previous studies have described the important role of family among Latinos in engaging in physical activity [37,45]. This finding is relevant because, as described by John et al. interventions that promote physical activity among Latinos “may be best served by targeting the family unit, integrating family-centric activities, and emphasizing the importance of social support and family communication to further leverage these important relationships to foster healthy behavior change” [46].

In this study, participants used the terms “physical activity” and “exercise” as synonymous. *Physical activity* is defined as any movement of the body based on skeletal muscle contraction resulting in a substantial increase in energy expenditure over resting levels [47]. *Exercise* is a specific, structured form of physical activity undertaken for improvement or maintenance of physical fitness. It is important that interventions that promote physical activity outline this difference. Physical activity includes all bodily movements that expend energy, including activities of daily living such as walking, occupational movement, household chores, and play. A person does not need to participate in structured, repetitive bodily movements (exercise) to be physically active and attain health benefits. This is important to note when working with individuals who do not engage in physical activity as exercise can be deterring for reasons including time, cost, and lack of skill or ability [48]. Using physical activity terminology may avoid that deterrence.

### Strengths and Limitations

Our study had several strengths. This study benefited from an adequate sample size that gave us sufficient information to understand the perspectives of Latinos who smoke on physical activity [49]. A strength of this study is the use of the HBM framework. This model provided a conceptual framework to successfully understand identified barriers, benefits, susceptibility, and cues to action. Another strength of this study is the heterogenicity of participants in regard to country of birth. The inclusion of Spanish-speaking participants is another study strength.

The study has several methodological limitations. First, data are self-reported and there is a possibility that participants felt compelled to offer socially desirable responses. Second, this study used a non-probability sampling method, which limits the generalizability of the findings. However, the community-based recruitment strategy employed in this study allowed us to reach a diverse sample of participants. Third, participants were not asked about their socioeconomic status (e.g., annual income, formal education) and type of occupation (e.g., physical or non-physical occupation). It is possible that these factors may impact their perspectives on physical activity. Lastly, participants were not asked about the use of other nicotine and tobacco products (e.g., electronic cigarettes), which is related to smoking behavior and should be included in future studies.

## 5. Conclusions

This qualitative study found multiple perceived factors of physical activity among Latino adults who smoke. These factors provide concrete operational strategies to address smoking cessation and physical activity among Latinos. Further research is needed on how best to integrate these perspectives into smoking cessation interventions.

## Figures and Tables

**Table 1:** Examples of the interview questions using the Health Belief Model.

Factor	Interview Questions
Perceived benefits	What are the benefits of being physically active?
Perceived susceptibility	What happens when someone is not physically active?
Perceived barriers	What makes it challenging for you to be physically active?
Cues to action	What motivates you to be physically active?

**Table 2:** Baseline characteristics of participants (n = 20).

Characteristics	n (%)
**Age**, Mean (SD)	54.9 (12.1)
**Gender,** n (%)	
Female	10 (50%)
Male	10 (50%)
**Sexual orientation,** n (%)	
Heterosexual or straight	17 (85%)
Homosexual or gay	1 (5%)
Prefer not to answer	2 (10%)
**Employment status,** n (%)	
Employed full-time	5 (25%)
Employed part-time	4 (20%)
Employed in more than one job	1 (5%)
Self-employed	1 (5%)
Student	1 (5%)
Unemployed	2 (10%)
Retired	1 (5%)
Unable to work or disabled	5 (25%)
**Language of preference,** n (%)	
Only Spanish	8 (40%)
More Spanish than English	6 (30%)
English and Spanish equally	5 (25%)
More English than Spanish	1 (5%)
Only English	-
**Country of birth,** n (%)	
USA	3 (15%)
Cuba	5 (25%)
Dominican Republic	5 (25%)
Mexico	2 (10%)
Puerto Rico	2 (10%)
Colombia	1 (5%)
Ecuador	1 (5%)
Venezuela	1 (5%)
**Cigarettes per day,** n (%)	
1–10 CPD	14 (70%)
11–20 CPD	3 (15%)
21 or more CPD	3 (15%)
**Time to first cigarette,** n (%)	
≤5 Minutes After Waking Up	7 (35%)
>5 Minutes After Waking Up	13 (65%)
**Use of menthol cigarettes,** n (%)	10 (50%)

**Table 3:** Participants’ age, gender, and language of preference.

Participant	Age	Gender	Language of Preference
Participant 1	62	Male	English and Spanish equally
Participant 2	60	Male	English and Spanish equally
Participant 3	43	Female	English and Spanish equally
Participant 4	54	Female	Only Spanish
Participant 5	72	Female	Only Spanish
Participant 6	46	Male	Only Spanish
Participant 7	59	Male	More English than Spanish
Participant 8	57	Female	More Spanish than English
Participant 9	61	Female	More Spanish than English
Participant 10	77	Male	Only Spanish
Participant 11	52	Female	Only Spanish
Participant 12	60	Male	More Spanish than English
Participant 13	59	Female	More Spanish than English
Participant 14	53	Male	Only Spanish
Participant 15	63	Female	More Spanish than English
Participant 16	53	Female	Only Spanish
Participant 17	40	Male	English and Spanish equally
Participant 18	61	Male	More Spanish than English
Participant 19	21	Male	English and Spanish equally
Participant 20	45	Female	Only Spanish

## Data Availability

The datasets generated for this study are available on request to the corresponding author.
